# Dystrophic neurites express C9orf72 in Alzheimer's disease brains

**DOI:** 10.1186/alzrt136

**Published:** 2012-08-16

**Authors:** Jun-ichi Satoh, Hiroko Tabunoki, Tsuyoshi Ishida, Yuko Saito, Kunimasa Arima

**Affiliations:** 1 Department of Bioinformatics and Molecular Neuropathology, Meiji Pharmaceutical University, 2-522-1 Noshio, Kiyose, Tokyo 204-8588, Japan; 2 Department of Pathology and Laboratory Medicine, Kohnodai Hospital, NCGM, 1-7-1 Kohnodai, Ichikawa, Chiba 272-8516, Japan; 3 Department of Laboratory Medicine, National Center Hospital, NCNP, 4-1-1 Ogawahigashi, Kodaira, Tokyo 187-8502, Japan; 4 Department of Psychiatry, National Center Hospital, NCNP, 4-1-1 Ogawahigashi, Kodaira, Tokyo 187-8502, Japan

## Abstract

**Introduction:**

Chromosome 9 open reading frame 72 (C9orf72) is an evolutionarily conserved protein with unknown function, expressed at high levels in the brain. An expanded hexanucleotide GGGGCC repeat located in the first intron of the C9orf72 gene represents the most common genetic cause of familial frontotemporal dementia (FTD) and amyotrophic lateral sclerosis (ALS). Previous studies by immunohistochemistry with two different anti-C9orf72 antibodies named sc-138763 and HPA023873 showed that C9orf72 is expressed chiefly in the cytoplasm of neurons, and is concentrated in the synaptic terminals in the brains of FTD/ALS with or without C9orf72 repeat expansion as well as those of controls. At present, a pathological role of C9orf72 in the process of neurodegeneration remains unknown.

**Methods:**

Using immunohistochemistry we studied C9orf72 expression in the frontal cortex and the hippocampus of six Alzheimer's disease (AD) and 13 control cases, including ALS, Parkinson's disease, multiple system atrophy, and non-neurological cases.

**Results:**

The HPA023873 antibody showed a cross-reactivity to glial fibrillary acidic protein, and therefore stained intensely reactive astrocytes in AD and non-AD brains. Both sc-138763 and HPA023873 antibodies labeled the neuronal cytoplasm and the neuropil with variable intensities, and intensely stained a cluster of p62-negative, UBQLN1-positive swollen neurites, which were distributed in the CA1 region and the molecular layer in the hippocampus of both AD and non-AD brains. Most notably, both of these antibodies reacted strongly with dystrophic neurites accumulated on senile plaques in AD brains.

**Conclusion:**

These results suggest a general role of C9orf72 in the process of neurodegeneration in a range of human neurodegenerative diseases.

## Introduction

Chromosome 9 open reading frame 72 (C9orf72) is an evolutionarily conserved protein with unknown function, expressed in most tissues including the brain. Recent studies indicate that an expanded hexanucleotide GGGGCC repeat located in the first intron of the C9orf72 gene represents the most common genetic abnormality for familial cases of frontotemporal dementia (FTD) and amyotrophic lateral sclerosis (ALS) with European ancestry, both of which constitute an overlapping continuum of a multisystem disorder affecting the central nervous system (CNS) [1-4]. The patients with the C9orf72 repeat expansion exhibit a clinical phenotype, characterized by an earlier disease onset with bulbar involvement, the presence of cognitive and behavioral impairment, psychosis, symmetrical frontotemporal atrophy, and reduced survival time [5-15]. The C9orf72 mutation is inherited in an autosomal dominant manner with incomplete penetrance. In contrast, the repeat expansion is found in less than 1% of Alzheimer's disease (AD) patients and normal subjects, and is extremely rare in Japanese ALS patients [14,16-18].

The noncoding C9orf72 repeats, expanding from 700 to 1,600 copies, inhibit the expression of one alternatively spliced transcript, and induce the formation of nuclear RNA foci composed of the hexanucleotide repeat [1]. The RNA foci sequester RNA-binding proteins, leading to aberrant mRNA splicing and processing of a set of genes pivotal for neuronal function [19]. The brains of FTD/ALS patients with the C9orf72 repeat expansion show not only the classical pathology, characterized by neuronal loss and astroglial and microglial activation prominent in the frontotemporal cortex, and degeneration of motor neurons in the spinal cord, but also the TAR DNA-binding protein-43 (TDP-43) pathology designated type B and/or type A most evident in the hippocampus [5-10]. Furthermore, numerous C9orf72-negative, TDP-43-negative, p62-positive neuronal cytoplasmic and nuclear inclusions are accumulated in the cerebellar granular cell layer and the dentate gyrus of the hippocampus of the brains of FTD/ALS patients with C9orf72 mutations [8,20]. Importantly, a panel of missense mutations is identified in the gene encoding p62, also known as sequestosome 1, in familial and sporadic ALS patients, supporting a key role for p62 in the pathogenesis of FTD/ALS [21].

By immunohistochemistry with two different commercially available anti-C9orf72 antibodies named sc-138763 and HPA023873, previous studies have shown that C9orf72 is expressed chiefly in the cytoplasm of neurons, presenting with varying immunoreactivities, and is highly concentrated in synaptic terminals in the neuropil [1,5-7,9,15]. Neuronal nuclei are largely devoid of C9orf72. In contrast, different studies have shown that C9orf72 is predominantly located in the nucleus of human fibroblasts and mouse NSC-34 motor neuron cells [2], and is expressed in both the cytoplasm and the nucleus of SH-SY5Y human neuroblastoma cells [3]. The discrepancy of subcellular location is attributable to differences in the cell types examined and the uncharacterized antibodies utilized. Importantly, no quantitative differences are observed in the levels of C9orf72 expression in the brains between FTD/ALS patients with or without C9orf72 repeat expansion and the controls, where intracellular inclusions except for Pick bodies do not express C9orf72 immunoreactivity [1-3,5-7,9,15]. On the contrary, the levels of C9orf72 protein are reduced in fibroblasts isolated from FTD/ALS patients with the repeat expansion [2].

At present, the physiological and pathological roles of C9orf72 in the CNS remain largely unknown, owing to a lack of thorough knowledge on C9orf72 expression and distribution in the human CNS. In the present study, we characterized the specificity of two anti-C9orf72 antibodies employed by previous studies. We found that the HPA023873 antibody shows a substantial cross-reactivity to glial fibrillary acidic protein (GFAP). We investigated the expression of C9orf72 in the frontal cortex and the hippocampus of six AD patients and 13 age-matched non-AD subjects by immunohistochemistry. We found that C9orf72 is expressed in dystrophic neurites accumulated on senile plaques of AD brains and focally swollen neurites distributed in the molecular layer in the hippocampus of both AD and non-AD brains, suggesting a general role of C9orf72 in the process of neurodegeneration.

## Materials and methods

### Human brain tissues

Serial sections of the frontal cortex and the hippocampus 10 μm thick were prepared from autopsied brains of six sporadic AD patients, comprising three men and three women with mean age 73 ± 9 years, and 13 non-AD patients, comprising six men and seven women with mean age 74 ± 8 years. The non-AD group included four normal subjects who died of non-neurological causes, three patients with sporadic Parkinson's disease (PD), four patients with sporadic ALS, and two patients with sporadic multiple system atrophy (MSA). The demographic profiles of the cases examined are shown in Table 1. All AD cases satisfied the Consortium to Establish a Registry for Alzheimer's Disease criteria for diagnosis of definite AD [22], and they were categorized into stage C of amyloid deposition and stage VI of neurofibrillary degeneration, following the Braak staging system [23].

**Table 1:** Demographic profile of the cases examined in the present study

Case	IHC	WB	Age (years)	Sex	Cause of death	Brain weight (g)	Postmortem interval (hours)	Braak staging (amyloid deposition/neurofibrillary degeneration)
NC1	+	+	88	F	Acute myocardial infarction	1,130	1.4	A/II
NC2	+	+	84	M	Acute myocardial infarction	1,350	1.6	0/II
NC3	+	+	77	M	Lung cancer	1,060	3.9	A/II
NC4	+	+	67	M	Dissecting aortic aneurysm	1,400	4.8	A/I
AD1	+	+	68	F	Pneumonia	1,000	1.1	C/VI
AD2		+	56	M	Pneumonia	1,230	14	C/VI
AD3	+	+	59	M	Pneumonia	1,220	10.5	C/VI
AD4	+	+	72	M	Pneumonia	1,240	8.1	C/VI
AD5	+	+	82	F	Lung cancer	1,090	4.5	C/VI
AD6	+	+	77	F	Pulmonary infarction	840	3	C/VI
AD7		+	70	M	Respiratory failure by aspiration	1,200	3.8	B/IV
AD8	+		80	M	Pneumonia	1,060	8	C/VI
PD1		+	86	F	Pneumonia	1,330	9.5	B/IV
PD2	+	+	83	F	Pneumonia	1,130	2.5	B/II
PD3	+	+	76	F	Respiratory failure by aspiration	910	2.5	B/II
PD4		+	56	M	Colon cancer	1,430	4	A/I
PD5	+		79	M	Pneumonia	1,320	9.3	C/III
ALS1	+	+	70	M	Respiratory failure	1,480	10.5	0/0
ALS2	+	+	75	F	Respiratory failure	1,090	1.3	0/I
ALS3	+	+	66	M	Respiratory failure	1,560	3	0/I
ALS4	+	+	61	F	Respiratory failure	1,320	10	0/II
ALS5		+	61	M	Respiratory failure	1,360	2.5	B/I
ALS6		+	74	M	Respiratory failure	1,600	13	B/I
MSA1	+		73	F	Pneumonia, septicemia	1,040	1.5	0/I
MSA2	+		66	F	Pneumonia	1,090	12	A/I

The demographic profile of the cases processed for immunohistochemistry (IHC) and western blotting (WB) is shown with the case number, age, sex, cause of death, brain weight, postmortem interval, and Braak Staging (amyloid deposition/neurofibrillary degeneration). AD, Alzheimer's disease; ALS, amyotrophic lateral sclerosis; F, female; M, male; MSA, multiple system atrophy; NC, non-neurological causes; PD, Parkinson's disease.

Autopsies on all subjects were performed at the National Center Hospital, National Center of Neurology and Psychiatry, Japan or at Kohnodai Hospital, National Center for Global Health and Medicine, Japan. The comprehensive examination of autopsied brains by three established neuropathologists (KA, YS, TI) validated the pathological diagnosis. Written informed consent was obtained from all cases. The Ethics Committee of the corresponding institutions approved the present study.

### Immunohistochemistry

The primary antibodies utilized in the present study and their working concentrations are presented in Table 2. The brain tissues were fixed with 4% paraformaldehyde and embedded in paraffin. After deparaffination, tissue sections were heat-treated in 10 mM citrate sodium buffer, pH 6.0 or pH 9.0, by autoclaving them at 125°C for 30 seconds in a temperature-controlled pressure chamber (Dako, Tokyo, Japan). The tissue sections were incubated at room temperature for 15 minutes with 3% hydrogen peroxide-containing methanol to block the endogenous peroxidase activity. For amyloid-beta immunolabeling, the sections were exposed to formic acid at room temperature for 5 minutes. They were incubated with PBS containing 10% normal goat or rabbit serum at room temperature for 15 minutes to block nonspecific staining. Subsequently, they were incubated at 4°C overnight with anti-human C9orf72 antibody raised against the peptide spanning amino acid residues 165 to 215 (sc-138763; Santa Cruz Biotechnology, Santa Cruz, CA, USA) or with anti-human C9orf72 antibody raised against the peptide spanning amino acid residues 110 to 199 (HPA023873; Sigma, St. Louis, MO, USA). In some experiments, the serial tissue sections were incubated with anti-ubiquilin-1 (UBQLN1; PLIC1) antibody (sc-14652; Santa Cruz Biotechnology) or anti-ubiquilin-2 (UBQLN2; PLIC2) antibody (sc-14658; Santa Cruz Biotechnology). The specificity of sc-14652 and sc-14658 was validated individually by western blot of the corresponding recombinant proteins expressed in HEK293 cells. We verified that the sc-14652 antibody does not label UBQLN2, while sc-14658 does not react with UBQLN1. After washing with PBS, the tissue sections were labeled at room temperature for 30 minutes with peroxidase-conjugated secondary antibodies (Nichirei, Tokyo, Japan), followed by incubation with diaminobenzidine tetrahydrochloride substrate (Vector, Burlingame, CA, USA). They were processed for a counterstain with hematoxylin. For negative controls, the primary antibody was omitted from the reaction.

**Table 2:** Primary antibodies utilized for immunohistochemistry and western blot analysis

Antibody	Supplier	Code	Origin	Antigen utilized for raising antibodies	Concentration used for IHC (μg/ml)	Concentration used for WB (μg/ml)
C9orf72	Santa Cruz Biotechnology (Santa Cruz, CA, USA)	sc-138763	Rabbit	Peptide spanning amino acid residues 165 to 215 of human C9orf72	0.1	0.1
C9orf72	Sigma (St. Louis, MO, USA)	HPA023873	Rabbit	Peptide spanning amino acid residues 110 to 199 of human C9orf72	0.72	0.1
UBQLN1	Santa Cruz Biotechnology	sc-14652	Goat	Peptide mapping within an internal region of human UBQLN1	1	0.2
UBQLN2	Santa Cruz Biotechnology	sc-14658	Goat	Peptide mapping within an internal region of human UBQLN2	2	NA
Amyloid beta 11 to 28	Immunobiological Laboratory (Gunma, Japan)	10027 (12B2)	Mouse	Human amyloid beta 11 to 28 peptide	1	NA
p62/sequestosome 1	BD Biosciences (San Jose, CA, USA)	610832	Mouse	Peptide spanning amino acid residues 257 to 437 of human p62	1	NA
Ubiquitin	Santa Cruz Biotechnology	sc-8017 (P4D1)	Mouse	Peptide spanning amino acid residues 1 to 76 of bovine ubiquitin	0.2	NA
PHF-tau	Thermo Scientific (Rockford, IL, USA)	MN1020 (AT8)	Mouse	Partially purified human PHF-tau	0.25	0.2
pS409/410 TDP-43	Cosomo Bio (Tokyo, Japan)	TIP-PTD-M01	Mouse	Phosphopeptide CMDSKpSpSGWGM	Diluted at 1:500	NA
GFAP	Nichirei (Tokyo, Japan)	422261 (GA5)	Mouse	GFAP purified from swine spinal cord	Prediluted	Further diluted at 1:1,000
HSP60 (N-20)	Santa Cruz Biotechnology	sc-1052	Goat	Peptide mapping in the N-terminus of human HSP60	NA	0.1

GFAP, glial fibrillary acidic protein; HSP60, 60 kDa heat shock protein; IHC, immunohistochemistry; NA, not applied; PHF, paired helical filament; TDP-43, TAR DNA-binding protein-43; WB, western blot; UBQLN, ubiquilin.

Double immunolabeling was performed according to the methods described previously [24]. The tissue sections were initially stained with anti-amyloid beta 11 to 28 antibody (12B2; Immunobiological Laboratory, Gunma, Japan), anti-p62 antibody (610832; BD Biosciences, San Jose, CA, USA), anti-ubiquitin antibody (sc-8017, P4D1; Santa Cruz Biotechnology), anti-phospho-TDP-43 antibody (pS409/410; Cosomo Bio, Tokyo, Japan), or anti-PHF-tau antibody (AT8; Thermo Scientific, Rockford, IL, USA). The staining was followed by incubation with alkaline phosphatase-conjugated secondary antibody (Nichirei), and colorization with New Fuchsin substrate (Nichirei). After inactivation of the antibodies by autoclaving the sections, they were relabeled with anti-C9orf72 antibody sc-138763 or HPA023873, and then were incubated with peroxidase-conjugated secondary antibodies, colorized with diaminobenzidine tetrahydrochloride substrate, and enhanced by exposure to diaminobenzidine tetrahydrochloride enhancing solution (Vector).

### RT-PCR analysis

[25]. Total cellular RNA was extracted using TRIZOL (Invitrogen, Carlsbad, CA, USA). RNA treated with DNase I was processed for cDNA synthesis using oligo(dT)_20 _primers and SuperScript II reverse transcriptase (Invitrogen). cDNA was then amplified by PCR using HotStar Taq DNA polymerase (Qiagen, Valencia, CA, USA) and a panel of sense and antisense primer sets: 5'-ccttgatttaacagcagagggcga-3' and 5'-tttccccacaccactgagctactt-3' for a 210 bp product specific for C9orf72 isoform a; 5'-gaatggaagatcagggtcacag-3' and 5'-gatggtatctgcttcatccagc-3' for a 221 bp product specific for C9orf72 isoform b; and 5'-ccatgttcgtcatgggtgtgaacca-3' and 5'-gccagtagaggcagggatgatgttc-3' for a 251 bp product of the G3PDH gene.

### Vector construction

To study the specificity of anti-C9orf72 antibodies, the full-length ORF of the human C9orf72 gene [GenBank:NM_018325] or the human GFAP gene [GenBank:NM_002055] was amplified by PCR using PfuTurbo DNA polymerase (Stratagene, La Jolla, CA, USA) and the set of sense and antisense primers. Subsequently, the PCR products were cloned in the expression vector pcDNA4/HisMax-TOPO (Invitrogen) to express a fusion protein with an N-terminal Xpress tag. The vectors were transfected in HEK293 cells using Lipofectamine 2000 reagent (Invitrogen) for transient expression.

### Western blot analysis

To prepare the total protein extract, cultured cells and frozen brain tissues were homogenized in RIPA buffer (Sigma) supplemented with a cocktail of protease inhibitors (Sigma), followed by centrifugation at 12,000 rpm for 10 minutes at room temperature to harvest the supernatant. The protein was separated on a 12% SDS-PAGE gel. After gel electrophoresis, the protein was transferred onto nitrocellulose membranes, followed by incubation at room temperature overnight with the anti-C9orf72 antibody sc-138763 or HPA023873. The membranes were then incubated at room temperature for 30 minutes with horseradish peroxidase-conjugated anti-rabbit IgG (Santa Cruz Biotechnology). The specific reaction was visualized by exposing the membranes to a chemiluminescent substrate (Pierce, Rockford, IL, USA). After the antibodies were stripped by incubating the membranes at 50°C for 30 minutes in stripping buffer, composed of 62.5 mM Tris-HCl, pH 6.7, 2% SDS and 100 mM 2-mercaptoethanol, the membranes were processed for relabeling with anti-GFAP antibody (GA5; Nichrei) or anti-heat shock protein Hsp60 antibody (sc-1052; Santa Cruz Biotechnology), which serves as an internal control of protein loading.

To prepare the total protein extract for two-dimensional gel electrophoretic analysis, the cells were homogenized in rehydration buffer composed of 8 M urea, 2% CHAPS, 0.5% carrier ampholytes pH 3 to 10, 20 mM dithiothreitol, 0.002% bromophenol blue, and a cocktail of protease inhibitors. Urea-soluble protein was separated by isoelectric focusing using the ZOOM IPGRunner system loaded with an immobilized pH 3 to 10 gradient strip (Invitrogen). After the first dimension of isoelectric focusing, the protein was separated in the second dimension on a 4 to 12% NuPAGE polyacrylamide gel (Invitrogen). The gel was then transferred onto a polyvinylidene difluoride membrane for western blot analysis.

### Statistical analysis

The signal intensity of C9orf72-immunopositive bands was quantified using ImageJ software (National Institute of Health, Bethesda, MD, USA), and was standardized individually by the signal intensity of Hsp60. The lysignificant difference between groups was evaluated by Student's *t *test. The correlation between GFAP and HSP023873 signals of individual cases was evaluated by Pearson's correlation coefficient test.

## Results

### Universal expression of C9orf72 mRNA in human neural cells

**Figure 1:**
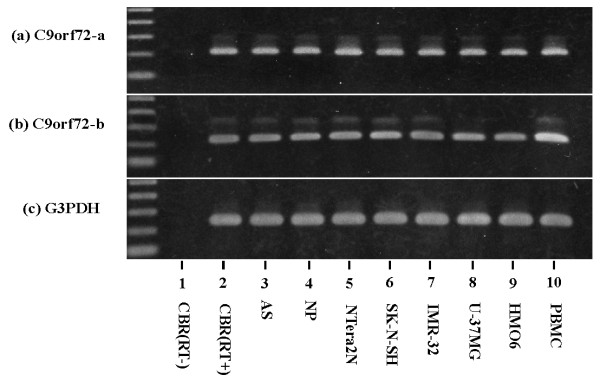
**Universal expression of C9orf72 mRNA in human neural cells**. Expression of the chromosome 9 open reading frame 72 (C9orf72) transcript encoding isoform a or isoform b was studied by RT-PCR in human tissues and cultured cells. **(a) **Isoform a. **(b) **Isoform b. **(c) **G3PDH, a housekeeping gene for a positive control. Lane 1, frontal cortex of the human cerebrum (CBR) without inclusion of the reverse transcription step; lane 2, CBR with inclusion of the reverse transcription step; lane 3, astrocytes (AS); lane 4, neuronal progenitor (NP) cells; lane 5, NTera2 teratocarcinoma-derived neurons; lane 6, SK-N-SH neuroblastoma; lane 7, IMR-32 neuroblastoma; lane 8, U-373MG glioblastoma; lane 9, HMO6 microglia; lane 10, peripheral blood mononuclear cells (PBMC).

The human C9orf72 gene encodes three distinct transcripts that produce two alternative isoforms. Transcript variants 3 [GenBank:NM_001256054] and 2 [GenBank:NM_018325] express a 481 amino acid protein encoded by exons 2 to 11 termed isoform a, while transcript variant 1 [GenBank:NM_145005] codes for a 222 amino acid protein encoded by exons 2 to 5 termed isoform b. Using RT-PCR, all of the cells and tissues - including the human cerebrum, astrocytes, neuronal progenitor cells, NTera2 teratocarcinoma-derived neurons, SK-N-SH neuroblastoma, IMR-32 neuroblastoma, U-373MG glioblastoma, HMO6 microglia, and peripheral blood mononuclear cells - expressed both isoforms (Figure 1a,b, lanes 2 to 10). The levels of G3PDH, a housekeeping gene, were almost constant in the cells and tissues examined (Figure 1c, lanes 2 to 10). No products were amplified when the reverse transcription step was omitted (Figure 1a to 1c, lane 1). The expression of mRNA coding for both isoforms a and b of C9orf72 is thus universal in human neural cells and peripheral blood mononuclear cells.

### Characterization of anti-C9orf72 antibodies

**Figure 2:**
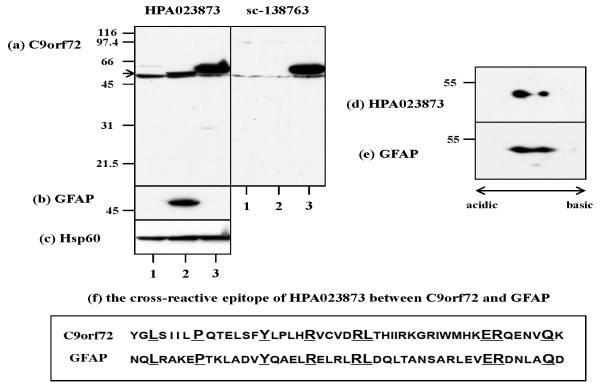
**Characterization of anti-C9orf72 antibodies**. Full-length ORF of the human chromosome 9 open reading frame 72 (C9orf72) gene or the human glial fibrillary acidic protein (GFAP) gene, cloned in the vector that expresses a fusion protein with an N-terminal Xpress tag, was transiently expressed in HEK293 cells. Total protein extract was processed for separation on one-dimensional (1D) or two-dimensional (2D) SDS-PAGE, followed by western blot analysis. **(a) **1D, C9orf72 labeled by HPA023873 (left) or sc-138763 (right). **(b) **1D, GFAP. **(c) **1D, Hsp60, an internal control for protein loading. **(d) **2D of the recombinant GFAP protein, HPA023873. **(e) **2D identical to (d), GFAP. Lane 1, nontransfected cells; lane 2, cells transfected with the vector expressing GFAP; lane 3, cells transfected with the vector expressing C9orf72. **(f) **A cross-reactive epitope between C9orf72 and GFAP recognized by HPA023873 aligned by the ClustalW program.

Before starting immunohistochemical studies, the specificity of two rabbit anti-human C9orf72 antibodies named sc-138763 and HPA023873 was verified by western blot analysis of the recombinant C9orf72 protein expressed in HEK293 cells. Both antibodies recognized a 58 kDa recombinant C9orf72 protein tagged with Xpress, in addition to a 54 kDa endogenous C9orf72 protein (Figure 2a, lanes 1 to 3). The bands of endogenous C9orf72 detected by HPA023873 were more intense than those labeled by sc-138763. In preliminary experiments, we found that HPA023873 but not sc-13873 stains intensely reactive astrocytes surrounding ischemic lesions in the brains of cerebral infarction by immunohistochemistry (data not shown). The reactivity of HPA023873 to GFAP was therefore determined by western blot analysis of the recombinant GFAP protein expressed in HEK293 cells. HPA023873 but not sc-138763 reacted with a 56 kDa GFAP protein tagged with Xpress (Figure 2a,b, lane 2; position of recombinant GFAP indicated by the arrow). The cross-reactivity of HPA023873 to GFAP was further determined by separation on two-dimensional SDS-PAGE of the recombinant GFAP protein, followed by western blot analysis (Figure 2d,e). These results indicated that both sc-138763 and HPA023873 antibodies reacted well with C9orf72, although the latter showed a substantial cross-reactivity to GFAP. The alignment of C9orf72 and GFAP amino acid sequences on ClustalW analysis [26] suggested the presence of a discontinuous epitope possibly responsible for the cross-reactivity within the immunogenic peptide of HPA023873 but not that of sc-138763 (Figure 2f).

### Western blot analysis of C9orf72 expression in human brain homogenates

**Figure 3:**
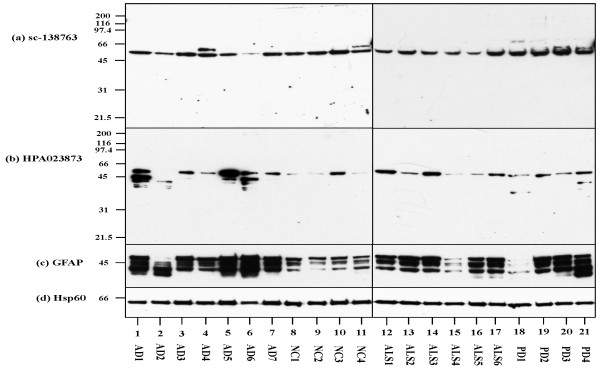
**C9orf72 expression in Alzheimer's disease and non-Alzheimer's disease brains by western blot analysis**. Expression of chromosome 9 open reading frame 72 (C9orf72) protein studied in the frozen frontal cortex tissues by western blot analysis. Total protein extract of 15 μg was loaded on each lane. **(a) **sc-138763. **(b) **HPA023873. **(c) **Glial fibrillary acidic protein (GFAP). **(d) **Hsp60, an internal control for protein loading. Lanes 1 to 7, brains derived from Alzheimer's disease (AD) patients; lanes 8 to 11, brains derived from normal control subjects (non-neurological cause (NC)); lanes 12 to 17, brains derived from amyotrophic lateral sclerosis (ALS) patients; and lanes 18 to 21, brains derived from Parkinson's disease (PD) patients (see Table 1). The position of the molecular weight marker is indicated on the left.

**Figure 4:**
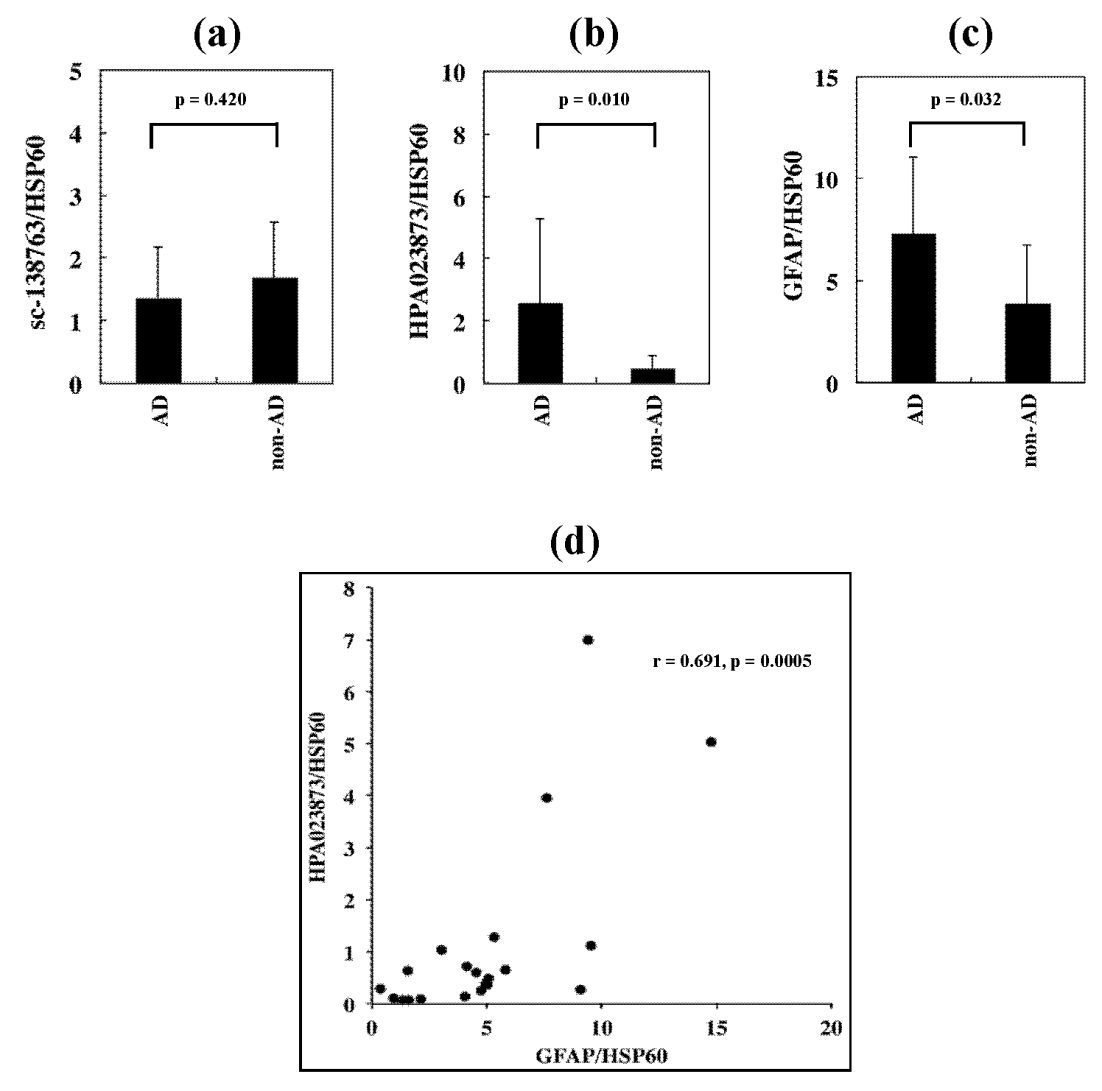
**Quantification of C9orf72 expression levels in Alzheimer's disease and non-Alzheimer's disease brains**. Expression of chromosome 9 open reading frame 72 (C9orf72) protein studied in the frozen frontal cortex tissues of seven Alzheimer's disease (AD) and 14 non-AD cases by western blot analysis as shown in Figure 3. Signal intensity of all immunopositive bands combined was quantified using ImageJ software, and was standardized individually by the signal intensity of Hsp60. Signal intensity ratio: **(a) **sc-138763/Hsp60, **(b) **HPA023873/Hsp60, and **(c) **glial fibrillary acidic protein (GFAP)/Hsp60. **(d) **Correlation between the GFAP/Hsp60 and HPA023873/Hsp60 ratios in individual cases.

Next, C9orf72 protein expression was studied in the frozen human frontal cortex tissues by western blot analysis. Both antibodies reacted well with a 54 kDa protein that corresponds to isoform a, expressed at variable levels among the brains of AD, ALS and PD patients and neurologically normal subjects (Figure 3a,b, lanes 1 to 21). We did not find expression of a 25 kDa protein corresponding to isoform b in any cases examined. The sc-138763 antibody reacted occasionally with a 58 kDa uncharacterized protein (Figure 3a, lanes 4 and 11). Furthermore, HPA023873 often labeled additional bands with molecular weight ranging from approximately 48 to 38 kDa (Figure 3b, lanes 1 to 3, 5, 6, 18 and 21). All of the brains expressed multiple GFAP isoforms with varying intensities (Figure 3c, lanes 1 to 21). By quantitative analysis, the levels of expression of sc-138763-immunoreactive bands were not different between AD and non-AD cases (Figure 4a), suggesting that the interindividual variation of sc-138763 immunoreactive bands is probably not disease specific. In contrast, the levels of expression of the bands immunopositive for HSP023873 and GFAP were elevated significantly in AD brains, compared with non-AD brains (Figure 4b and 4c). Notably, there existed a positive correlation between the levels of GFAP and those of HSP023873 in individual cases (*r *= 0.691, *P *= 0.0005) (Figure 4d), consistent with the observation that HPA023873 exhibits cross-reactivity to GFAP.

### Immunohistochemical characterization of C9orf72 expression in AD and non-AD brains

**Figure 5:**
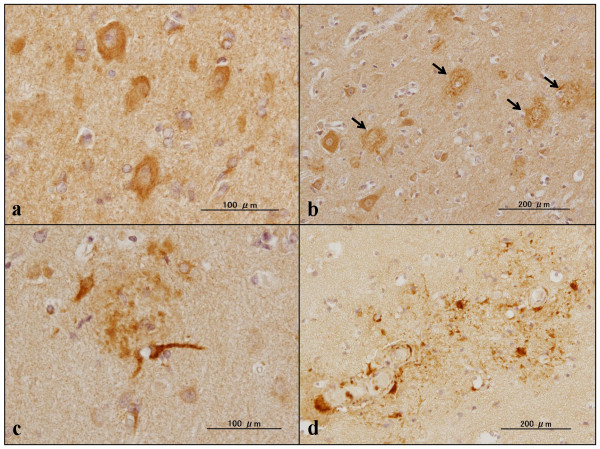
**C9orf72 immunoreactivity in the frontal cortex of Alzheimer's disease brains**. Expression of chromosome 9 open reading frame 72 (C9orf72) studied in the frontal cortex of Alzheimer's disease brains by immunohistochemistry using sc-138763 and HPA023873 antibodies. **(a) **sc-138763, neurons and the neuropil. **(b) **HPA023873, neurons, the neuropil, and senile plaques (arrows). **(c) **HPA023873, senile plaque and surrounding astrocytes. **(d) **HPA023873, perivascular plaques with reactive astrocytes.

**Figure 6:**
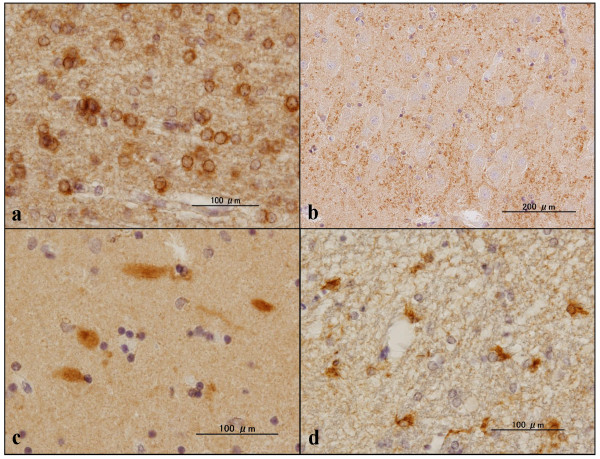
**C9orf72 immunoreactivity in the hippocampus of non-Alzheimer's disease brains**. Expression of chromosome 9 open reading frame 72 (C9orf72) studied in the hippocampus of non-Alzheimer's disease brains by immunohistochemistry using sc-138763 and HPA023873 antibodies. **(a) **sc-138763, Parkinson's disease (PD), oligodendrocytes in the white matter. **(b) **sc-138763, normal subject, synaptic terminals in the neuropil of CA2. **(c) **sc-138763, PD, swollen dystrophic neurites with stick and rugby ball shapes in the molecular layer. **(d) **HPA023873, PD, reactive astrocytes in the periventricular white matter.

**Figure 7:**
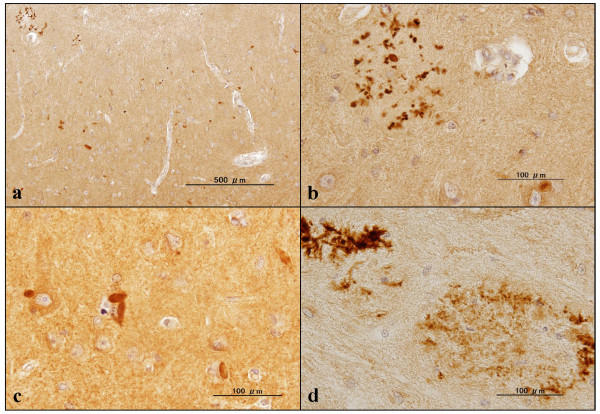
**C9orf72 immunoreactivity in the hippocampus of Alzheimer's disease brains**. Expression of chromosome 9 open reading frame 72 (C9orf72) studied in the hippocampus of Alzheimer's disease brains by immunohistochemistry using sc-138763 and HPA023873 antibodies. **(a) **sc-138763, CA1 overview. **(b) **sc-138763, CA1, dystrophic neurites accumulated on senile plaque. **(c) **sc-138763, CA1, dystrophic neurites with stick and rugby ball shapes. **(d) **HPA023873, CA4, dystrophic neurites accumulated on senile plaques.

**Figure 8:**
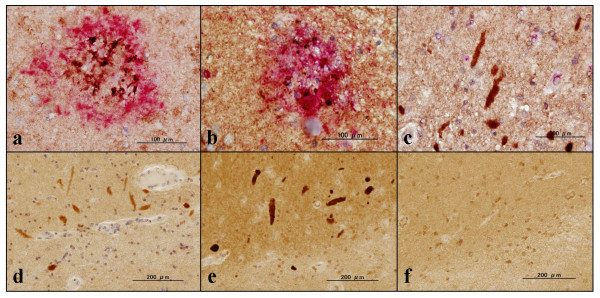
**C9orf72, amyloid beta, PHF-tau, p62, UBQLN1, and UBQLN2 immunoreactivities in Alzheimer's disease and non-Alzheimer's disease**. Expression of chromosome 9 open reading frame 72 (C9orf72), amyloid beta (Aβ), PHF-tau, p62, ubiquilin (UBQLN)1, and UBQLN2 studied in the hippocampus of Alzheimer's disease (AD) and non-AD brains by double immunolabeling or single labeling of the serial sections. **(a) **HPA023873 (brown) and red (Aβ), AD, CA4, C9orf72-positive dystrophic neurites on amyloid plaques. **(b) **sc-138763 (brown) and AT8 (red), AD, C9orf72-positive PHF-tau-positive dystrophic neurites on senile plaques on the molecular layer. **(c) **sc-138763 (brown) and p62 (red), multiple system atrophy (MSA), C9orf72-positive p62-negative swollen dystrophic neurites in the molecular layer. **(d) **sc-138763 single labeling, MSA, C9orf72-positive swollen dystrophic neurites in the molecular layer. **(e) **UBQLN1 single labeling, MSA, area identical to (d), swollen dystrophic neurites positive for UBQLN1. **(f) **UBQLN2 single labeling, MSA, area identical to (d), swollen dystrophic neurites negative for UBQLN2.

Finally, expression of C9orf72 was studied in the frontal cortex and the hippocampus of six AD patients and 13 non-AD cases by immunohistochemistry. The sc-138763 antibody stained the cell bodies of neurons and oligodendrocytes, labeled with variable intensities in both AD and non-AD brains (Figures 5a,b and 6a). The neuronal and oligodendroglial nuclei were always devoid of sc-138763 immunoreactivity. The neuropils in the hippocampus CA2 and CA3 regions frequently showed a coarse punctuate sc-138763 immunoreactivity suggestive of location at synaptic terminals in both AD and non-AD brains (Figure 6b). In contrast, sc-138763 did not react with astrocytes or microglia in any brains examined. Notably, the sc-138763 antibody intensely labeled a cluster of focally swollen, dystrophic neurites with irregular-shaped stick-like and rugby ball-like morphologies, which were mostly distributed in the CA1 region and the molecular layer in the hippocampus of AD, ALS, PD, MSA, and normal control brains (Figures 6c and 7a,c). Most notably, sc-138763 reacted strongly with dot-like, button-like and string-like dystrophic neurites positive for PHF-tau (AT8) accumulated on senile plaques (Figures 7b and 8b).

The HPA023873 antibody stained the cell bodies of neurons and reactive astrocytes, and the neuropil, labeled with variable intensities, but labeled neither oligodendrocytes nor microglia, in both AD and non-AD brains (Figure 6d). Most notably, HPA023873 intensely stained not all but considerable numbers of senile plaques with or without accumulation of dystrophic neurites, and surrounding astrocytes in AD brains (Figures 5c,d, 7d and 8a). In contrast, HPA023873 reacted barely with swollen dystrophic neurites recognized by sc-138763 distributed in the CA1 region and the molecular layer of the hippocampus in AD and non-AD brains. By double immunolabeling or single labeling of the serial sections, swollen dystrophic neurites labeled by sc-138763 were mostly negative for p62, ubiquitin, UBQLN2, phospho-TDP-43 and PHF-tau, but positive for UBQLN1 (Figure 8c to f). Furthermore, both sc-138763 and HPA023873 antibodies did not stain any type of cytoplasmic and nuclear inclusions in neurons and glial cells if they exist in AD and non-AD brains. We detected no cross-reactivity of anti-UBQLN1 (sc-14652) antibody and anti-PHF-tau (AT8) antibody to GFAP by western blot (data not shown).

## Discussion

The human C9orf72 gene encodes a 54 kDa protein with unknown function, expressed at high levels in the CNS. Previous studies by immunohistochemistry with two different anti-C9orf72 antibodies named sc-138763 and HPA023873 showed that C9orf72 is expressed chiefly in the cytoplasm of neurons, and is highly concentrated in the synaptic terminals in the brains of FTD/ALS with or without C9orf72 repeat expansion as well as those of controls [1,5-7,9,15]. In addition, these antibodies did not react with any intracellular inclusions except for Pick bodies in the disease-affected brains [1-3,5-7,9,15]. Finally, a recent study concluded that C9orf72 immunolabeling of FTD, ALS, AD, and control brains with HPA023873 could not identify the disease-specific pathology [27].

In the present study, we found by RT-PCR that the transcripts encoding C9orf72 isoforms a and b are expressed widely in human neural cells. We characterized the specificity of sc-138763 and HPA023873 antibodies, and found that both antibodies react well with C9orf72 - but HPA023873 exhibits a substantial cross-reactivity to GFAP, and therefore intensely stains reactive astrocytes in both AD and non-AD brains. The sc-138763 antibody was raised against the peptide mapping within amino acid residues 165 to 215 of C9orf72, while HPA023873 was directed to residues 110 to 199 containing the GFAP cross-reactive epitope spanning residues 112 to 155 (Figure 2f). Currently, another two rabbit anti-C90rf72 antibodies are commercially available: GTX119776 directed to residues 1 to 198 (GeneTex, Irvine, CA, USA) and 22637-1-AP directed to residues 1 to 169 (ProteinTech, Chicago, IL 60612, USA), both of which share the putative GFAP cross-reactive epitope.

By western blot analysis we identified the expression of C9orf72 protein in the brains of AD, ALS, and PD patients and normal subjects, with the levels of expression showing a possibly disease-nonspecific interindividual variation, although we have not yet attempted to determine C9orf72 mutations in any cases examined. However, a recent nationwide study showed that C9orf72 mutations are extremely rare in Japanese familial and sporadic ALS patients, where two patients out of a total 563 ALS patients (0.4%) exhibited the C9orf72 repeat expansion [18]. By immunohistochemistry we found that both antibodies stained the neuronal cytoplasm and the neuropil labeled with variable intensities. Furthermore, antibodies, much more intensely sc-138763, labeled a cluster of p62-negative, UBQLN1-positive swollen dystrophic neurites distributed in the CA1 region and the molecular layer in the hippocampus of both AD and non-AD brains. Most notably, both sc-138763 and HPA023873 antibodies reacted strongly with not all but substantial numbers of dystrophic neurites accumulated on senile plaques in AD brains. These observations suggest a general role of C9orf72 in the process of neurodegeneration in a range of human neurodegenerative diseases.

Using anti-UBQLN2 antibodies named 5F5 (Abnova, Walnut, CA, USA) and AP12092PU-N (Acris, San Diego, CA, USA), a recent study showed that numerous immunopositive aggregates and dystrophic neurites are accumulated in the hippocampal molecular layer and CA1-CA4 regions in the brains of FTLD/ALS patients with C9orf72 expansion, whereas the brains of the cases without the expansion barely show these structures [27]. However, both of these antibodies are generated against the peptides, whose amino acid sequences are shared mostly between UBQLN1 and UBQLN2. These antibodies therefore could not discriminate immunoreactivities between UBQLN1 and UBQLN2. In contrast, we utilized anti-UBQLN1 antibody sc-14652 and anti-UBQLN2 antibody sc-14658, whose specificities were individually validated by western blot analysis of the corresponding recombinant proteins expressed in HEK293 cells. We verified that sc-14652 does not label UBQLN2, whereas sc-14658 does not react with UBQLN1.

Increasing evidence indicates that both UBQLN1 and UBQLN2 play a central role in the ubiquitin/proteasome system that degrades short-lived and misfolded ubiqutinated proteins, while p62 acts mainly as a cargo receptor for selective autophagy that degrades larger structures, including protein aggregates or entire organelles [28,29]. p62 is found to be co-localized with ubiquitin in neuronal and glial inclusions in AD, PD, and MSA brains [30]. In contrast, we did not identify C9orf72-immunopositive neuronal and glial inclusions in any brains examined. UBQLN1, by acting as an interactor for presenilin-1 and presenilin-2, promotes accumulation of presenilin proteins [31]. Missense mutations in the UBQLN2 gene are identified in some cases of dominantly inherited, chromosome-X-linked ALS with dementia [32]. In the present study using the ubiquilin class-specific antibodies, focally swollen, dystrophic neurites distributed in the CA1 region and the molecular layer in the hippocampus of both AD and non-AD brains express both UBQLN1 and C9orf72, but they do not express phospho-TDP-43, p62 or UBQLN2, suggesting the possibility that C9orf72 concentrated in dystrophic neurites plays a key role in the homeostasis of protein degradation, by acting in cooperation with UBQLN1.

## Conclusion

C9orf72 is expressed in dystrophic neurites accumulated on senile plaques in AD brains, and in swollen dystrophic neurites distributed in the CA1 region and the molecular layer in the hippocampus of AD, ALS, PD, MSA, and normal control brains. These results suggest a more general role for C9orf72 in the process of neurodegeneration in various human neurodegenerative diseases. This view should be further evaluated by studies on large-scale samples of AD and other neurodegenerative diseases with highly specific antibodies.

## Abbreviations

AD: Alzheimer's disease; ALS: amyotrophic lateral sclerosis; bp: base pair; C9orf72: chromosome 9 open reading frame 72; CNS: central nervous system; FTD: frontotemporal dementia; G3PDH: glyceraldehyde-3-phosphate dehydrogenase; GFAP: glial fibrillary acidic protein; HSP: heat shock protein; MSA: multiple system atrophy; ORF: open reading frame; PBS: phosphate-buffered saline; PCR: polymerase chain reaction; PD: Parkinson's disease; PHF: paired helical filament; RT: reverse transcriptase; TDP-43: TAR DNA-binding protein-43; UBQLN, ubiquilin.

## Competing interests

The authors declare that they have no competing interests.

## Authors' contributions

J-IS and HT carried out western blot and immunohistochemistry analysis, and drafted the manuscript. TI, YS, and KA validated the pathological diagnosis of autopsied brains. All authors read and approved the final manuscript.
